# Present and future distribution of bat hosts of sarbecoviruses: implications for conservation and public health

**DOI:** 10.1098/rspb.2022.0397

**Published:** 2022-05-25

**Authors:** Renata L. Muylaert, Tigga Kingston, Jinhong Luo, Maurício Humberto Vancine, Nikolas Galli, Colin J. Carlson, Reju Sam John, Maria Cristina Rulli, David T. S. Hayman

**Affiliations:** ^1^ Massey University, Palmerston North, New Zealand; ^2^ Texas Tech University, Lubbock, TX, USA; ^3^ Central China Normal University, Wuhan, People's Republic of China; ^4^ São Paulo State University, Rio Claro, Brazil; ^5^ Politecnico di Milano, Milan, Italy; ^6^ Georgetown University Medical Center, Washington, DC, USA

**Keywords:** ecological niche models, climate change, SARS-like coronavirus, forecasting, diversity

## Abstract

Global changes in response to human encroachment into natural habitats and carbon emissions are driving the biodiversity extinction crisis and increasing disease emergence risk. Host distributions are one critical component to identify areas at risk of viral spillover, and bats act as reservoirs of diverse viruses. We developed a reproducible ecological niche modelling pipeline for bat hosts of SARS-like viruses (subgenus *Sarbecovirus*), given that several closely related viruses have been discovered and sarbecovirus–host interactions have gained attention since SARS-CoV-2 emergence. We assessed sampling biases and modelled current distributions of bats based on climate and landscape relationships and project future scenarios for host hotspots. The most important predictors of species distributions were temperature seasonality and cave availability. We identified concentrated host hotspots in Myanmar and projected range contractions for most species by 2100. Our projections indicate hotspots will shift east in Southeast Asia in locations greater than 2°C hotter in a fossil-fuelled development future. Hotspot shifts have implications for conservation and public health, as loss of population connectivity can lead to local extinctions, and remaining hotspots may concentrate near human populations.

## 1. Introduction

Major current and future global changes pose a severe risk to biodiversity and human survival [1]. Global climate change and human encroachment into natural habitats are simultaneously driving the biodiversity extinction crisis and increasing disease emergence risk [2]. Climate and land cover change will alter the distribution of species [3], an important but poorly defined predictor of zoonotic disease risk [4,5]. The direction and magnitude of range shifts are not estimated for many species, leaving the impacts on their viral interactions uncertain [6,7].

In early 2020, genomic analysis identified the severe acute respiratory syndrome coronavirus 2 (SARS-CoV-2), responsible for the coronavirus disease 2019 (COVID-19) pandemic. SARS-CoV-2 is closely related to viruses present in the intermediate horseshoe bat *Rhinolophus affinis* (virus RaTG13, sampled from the Yunnan province of China in 2013 [8]), and the following bats captured in northern Lao People's Democratic Republic (hereafter, Lao PDR) in 2020: *Rhinolophus malayanus* (virus RmYN02), *Rhinolophus marshalli* (virus BANAL-236) and *Rhinolophus pusillus* (virus BANAL-103) [9]). These viruses, like SARS-CoV-2, are part of the *Sarbecovirus* subgenus, and belong to the *Betacoronavirus* genus (family *Coronaviridae*, subfamily *Orthocoronavirinae*). In the year since SARS-CoV-2 was described, 47 potential *Betacoronavirus* hosts have been reported [10]. Knowledge about hosts and where they are gives insights into emerging disease origins and clues on future risk [11,12].

Bats comprise approximately 20% of global mammal taxonomic diversity, with more than 1435 species described [13], among 6490 known extant mammals [14]. This diversity probably contributes to the viral diversity in bats [15,16], including some viruses that have emerged as pathogenic in people [17,18], such as sarbecoviruses [12]. Bats have multiple functions within ecosystems, acting as pollinators, seed dispersers and insect predators [19]. However, over a fifth of species are Threatened or Near Threatened with extinction according to the IUCN Red List 2021 (www.iucnredlist.org) and drivers of changes in bat distributions, such as land-use change, probably contribute both to population declines and the simultaneous increase in infectious disease emergence risk [11,20]. Improved range estimates can, therefore, support conservation strategies and understanding disease risk.

Among factors influencing bat distributions, suitable climatic limits and karst are critical for many species [21–23]. The presence of native habitat, especially dense forest is also vital for many species [3,24]. Maintaining such habitats has important implications for conservation and potentially viral transmission through changes in species interactions and survival probability [7]. For example, host dispersal among vampire bats, and specifically males, has facilitated rabies spread in Peru [25] and sympatry has led to host shifts among bat coronaviruses [26,27]. There are, however, knowledge gaps ranging from bat distributional ecology to their behaviour, immunity and physiology [16].

Attempts to estimate bat sarbecovirus-host spatial distributions have included modelling near-current distributions for bats of the family Rhinolophidae in Southeast Asia [28] and filtering their expected areas of habitat [12]. However, estimating species distributions with future projections can help us understand their conservation status, inform land-use planning to avoid conflicts [29], generate better models for estimating the risk of emerging novel pathogens, and allow targeted infectious disease surveillance. The rapid increase in bat data after the COVID-19 pandemic provides opportunities to better understand bats' distributional ecology, but may bring sampling biases in areas where surveillance has been greatest [11,30,31]. Avoiding misprediction is essential, and we have the opportunity to update ecological niche models with the help of big data, reproducible tools and open science. We need adequate inferences regarding bat species distributions from the current period projected to proximate future scenarios, so we can establish guidelines for how to transition from the current trajectory of biodiversity loss and pandemic risk to a more sustainable future [1].

Here, we use ecological niche models to assess the potential distribution of bat hosts to *Sarbecovirus* in order to address the following questions. (a) What is the availability and spatial coverage of data for inferring the distribution of bats known to host sarbecoviruses? (b) How are *Sarbecovirus* bat host distributions affected by climate, karst and forest amount in the near-current and future scenarios? (c) Where are current and future areas with high species richness (hotspots) of *Sarbecovirus* bat hosts? Finally, we share a dynamic data analysis pipeline, considering the inevitable addition of new data on host species in the future.

## 2. Methods

### (a) Target species and occurrence data

All analyses were in R v. 4.1.2 [32] (electronic supplementary material, figure S1). Code and workflow [33] are provided in GitHub (https://github.com/renatamuy/dynamic) and data in Dryad [34]. We spatially predicted the occurrence of all known *Sarbecovirus* hosts regardless of the first viral detection location using Ecological Niche Models (ENMs) and approximating them to species distribution models (SDMs). We compiled host data (electronic supplementary material, table S1) from published articles, preprints and NCBI (National Center for Biotechnology Information) accession numbers cross-checked in Virion v. 0.2.1 [35] with Genbank references. Bat hosts of *Sarbecovirus* viruses were: (1) explicitly named in the reference; and (2) the source of viruses or viral fragments of *Sarbecovirus* or synonyms for SARS-related coronaviruses.

Then, in September 2021 we mined bat host species occurrences from: Darkcides v1 [23], Global Biodiversity Information Facility (GBIF) [36], Berkeley Ecoinformatics Engine (Ecoengine), Vertnet, Integrated Digitized Biocollections (IDigBio), iNaturalist, Obis and data compiled for previous publications [11,37]. We filtered all data sources, and with iNaturalist kept only ‘research quality grade’ data. We performed data mining using a custom loop through spocc function [21]. We only kept records from 1970 onwards, and records from 1970 to 2000 comprised only 4% of records. All points from DarkCideS (*n* = 1351) are from the 2000s onwards.

### (b) Sampling bias assessment

To reduce spatial sampling bias due to uneven and undersampling [38,39], we performed a series of filtering steps, removing data concentrated in political centroids of countries, provinces, national capitals and centroids for GBIF headquarters and museums. Duplicate coordinates for the same species and points in permanent water bodies and oceans were excluded with clean_coordinates from CoordinateCleaner 2.0-18 [40] and we used cc_outl to remove geographical outliers, defined by the interquartile range. Because the number of points can define outliers differently and lead to information loss for rare data, we set 20 as the minimum value for correction. We then visually inspected points in QGIS 3.10.7 [41].

We inspected points for taxonomic and range consistency based on IUCN Red List polygons and the *Handbook of the Mammals of the World* when an IUCN polygon was not available [42]. We used IUCN Red List habitat and population trend information to discuss our findings. For species complexes, the broader range matching the viral detection information was intersected with input data points. Species complexes like *Hipposideros pomona* and *H. ruber* were treated as *sensu lato* across their distributions. For *H. pomona*, we considered the broad distribution for *H. gentilis* as a mask for filtering occurrences for the IUCN-intersected input. *H. ruber* occurrences were kept within and outside their matching IUCN polygons, since range and taxonomy may need review. Occurrences of *Rhinolophus cornutus* in continental areas from DarkCides were not considered, as they refer to *R. pusillus* in recent assessments [43].

Final preprocessing point thinning was made in ENMTML [44], using an approximated search-cell radius of 13 km. We did not perform further environmental filtering as the largest gains in model performance using those filters are for 10 points or fewer [45] and we only ran models with more than 40 points, as is good practice [46].

We assume that areas with low sampling coverage driven by accessibility bias should be investigated more, especially if SDMs predict a suitable habitat for a high numbers of species. We assessed accessibility bias for the thinned occurrence data (regardless of species) considering the distance of points from cities, rivers, roads and airports through sampbias [47]. We estimated how sampling rates (a Poisson process with rate *λ*) vary as a function of proximity to drivers, generating a sampling coverage metric driven by accessibility bias. After generating the layers including estimated sampling rates (input parameters in electronic supplementary material, table S2), we used a bivariate choropleth map to visualize which highly species-rich areas predicted by our ensembles have high values of estimated sampling rates.

### (c) Accessible area and spatial restriction

Accessible areas where species may disperse were defined by the extent of the ecoregion [48] within which each species occurred (IUCN polygon intersected and non-intersected). This extent was used for geographically and environmentally constrained background point sampling [49] with the default ratio between presence and background points around a 50 km buffer from occurrence points. Data partitioning for training:testing was 75 : 25% by split sample. We included dispersal capability in the model ensembles using the *a posteriori* method ‘OBR’ for SDMs. This method was coupled within the accessible areas for spatial restriction of the final maps, as it performed well in virtual species tests [50], reducing overprediction without increasing omission errors. Because the ‘OBR’ method [50] is not applied to future projections (we cannot restrict them based on observed future occurrences), we compared all projected ranges within the accessible area in future scenarios as being the maximum limit for dispersal. We define range as the area where the species most likely occurs (estimated occupied area) driven by the environmental covariates used.

### (d) Covariates and workflow

We defined environmental variables that are important drivers of target species distributions in the current geographical space, which can also be projected into the future. Based on the focal species' ecology, we chose selected climatic (annual precipitation, precipitation seasonality, annual mean temperature, temperature seasonality) and landscape variables (karst and primary forest cover) as covariates (electronic supplementary material, table S3).

Habitats used by each species were extracted using rredlist v. 0.7 (electronic supplementary material, figure S2). We selected forest cover as the main land-cover variable to avoid correlation and error inflation due to limited data. Furthermore, most of our target species benefit from forest physiognomies (electronic supplementary material, figure S2). Forest habitat was calculated as a proportion from LUH2 [51] for near-current and future scenarios at 0.25 dd, so we resampled all other layers through bilinear interpolation to match this resolution. Distance to cave or karst was the only static variable. We calculated the minimum euclidean distance (km) from karst or cave and averaged it within the working resolution grid. Distances were calculated in QGIS 3.10.7 after warping layers to Mercator metric projection Datum WGS84 and then reprojected back to the geographical system with Datum WGS84.

Climate predictors were downloaded from WorldClim 2.1 [52] with 10 arc-minutes spatial resolution. We selected covariates with Pearson's product-moment correlation value |*r*| < 0.70 [53] across all covariates prior to modelling to avoid collinearity (electronic supplementary material, figure S3). From 19 near-current (1970–2000) bioclimatic variables, we selected annual mean temperature (bio_1), temperature seasonality (standard deviation × 100, bio_4), annual precipitation (bio_12), and precipitation seasonality (coefficient of variation, bio_15).

We built ENM ensembles using ‘MXS’ and ‘MXD’ maximum entropy algorithms. We selected these algorithms based on experience and performance [50,54,55]. We used consensus ensemble maps including the suitability values weighted by TSS (weighted mean of True Skill Statistics values) and report performance using TSS, but also report Boyce discrimination values [56]. Each model algorithm was replicated 10 times through the ‘bootstrap’ term in ENMTML. Despite being called bootstrap, this method applies a split sampling method. To evaluate model performance, we randomized occurrence data into 75% : 25% train:test samples to calculate the TSS [34] for each model. Models with TSS > 0.5 were considered as performing above that expected by chance [57]. We weighted the ensembles based on model performance and used weighted TSS value differences for selecting the most realistic maps between IUCN-intersected and non-IUCN-intersected data. Threshold values were calculated to transform each model's predictions to presence or absence of each species, using ‘MAX_TSS’, the threshold at which the sum of the sensitivity and specificity is the highest [58]. After generating species maps, we calculated host richness as the sum of presences of each host per pixel in the binary maps, producing a map of host–species taxonomic richness. We derived zonal statistics based on the host species map for the country's shapefile, calculating the maximum predicted host richness per country using administrative regions from Natural Earth (https://www.naturalearthdata.com/). We compared estimated taxonomic richness maps generated from the two sets of models to check for convergent patterns using Pearson's product-moment correlation. We ran models for two sets of filtered and thinned datasets: (1) IUCN-intersected polygon data; and (2) non-IUCN polygon intersected data. We compared performance values between those two sets of ensembles to infer improvement in performance. Correlative variable contribution was inspected throughout each set of model algorithms.

### (e) Hotspots and future projections

We downloaded SSP (Shared Socioeconomic Pathways) scenarios for Coupled Model Intercomparison Project Phase 6 (CMIP6) at 10 arc-minutes spatial resolution. We present results for projections using the SSP2-4.5 and SSP5-8.5, focusing on SSP5-8.5 for our results. SSPs represent baseline paths with varying human behaviour reflected in actions changing land cover, and coupled with greenhouse gas (GHG) emissions they provide future global change scenarios. Scenario SSP5-8.5, for instance, means 8.5 radiative forcing level coupled with an SSP5 (fossil-fuelled development), which translates in a pessimistic scenario [59]. The SSP2-4.5 is known as a middle-of-the-road scenario. Here, we present results for BCC-CSM2-MR (Beijing Climate Center Climate System Model) and CanESM5 (Canadian Earth System Model v. 5) GCMs for 2021–2040, 2041–2060, 2061–2080, 2081–2100 periods. We report values for SSP5-8.5 and BCC-CSM2-MR for 2100 in our maps, as a more extreme, but possible future scenario. We include results for all scenarios, periods and GCMs in the supplements. Our pipeline easily incorporates all combinations of SSPs, periods and GCMs, depending on computational power. We resampled all future rasters to 0.25 dd using the bilinear method. Area calculations of range shifts and shifts in range overlap were made for each consensus binary map. Hotspots were simply defined as pixels with the greatest predicted species richness. The centroids of hotspots in the present and future were calculated to describe changes in climate and their locations. To investigate if hotspots were getting physically hotter we inspected the distribution of richness values and average temperature in the present and by 2100 for SSP5-8.5 and SSP2-4.5. Finally, changes in the number of contiguous areas with the highest values of estimated richness and division index were calculated with landscape metrics [60].

## 3. Results

Sarbecoviruses were reported from 35 bat species (electronic supplementary material, table S1). We could model the potential distribution of 17 species using IUCN-intersected data, and 23 with non-intersected data (electronic supplementary material, figure S4). Of the latter 23 species, the potential distribution of six could only be modelled without intersecting their points with IUCN data; models for nine species did not show improvement in TSS values after intersecting data with IUCN ranges, while for eight there were small improvements after cropping occurrences within IUCN range limits (electronic supplementary material, table S4).

The maps show three focal areas of suitability across species; one each in Western Europe, Indochina and Central Africa (electronic supplementary material, figures S5 and S6). IUCN delimitation for occurrence inclusion does not improve model performance for more than 10% added value in TSS in most cases (electronic supplementary material, table S4). Richness maps for the two datasets were highly positively correlated (|*r*| 0.955; *p*-value < 0.0001), indicating agreement between richness hotspots for IUCN-intersected data and data gathered inside and outside IUCN polygons. Therefore, we report the non-IUCN intersected datasets. Overall, species with smaller ranges had fewer filtered points (electronic supplementary material, figure S7).

Environmental covariates affected *Sarbecovirus* bat hosts differently, with temperature seasonality (*n* = 12), karst (*n* = 5) or precipitation seasonality (*n* = 4) the top-ranked variables for most species considering the correlative covariate importance, with all variables varying in their relative contribution (electronic supplementary material, figure S8). All variables had importance values above 26% at least once, depending on the species. From the lower ranking contributors, annual precipitation had greater than 10% contribution for 19 of 23 species (82%), annual mean temperature greater than or equal to 10% for 14 (61%), and forest amount greater than 10% for ten (43%).

**Figure 1.**
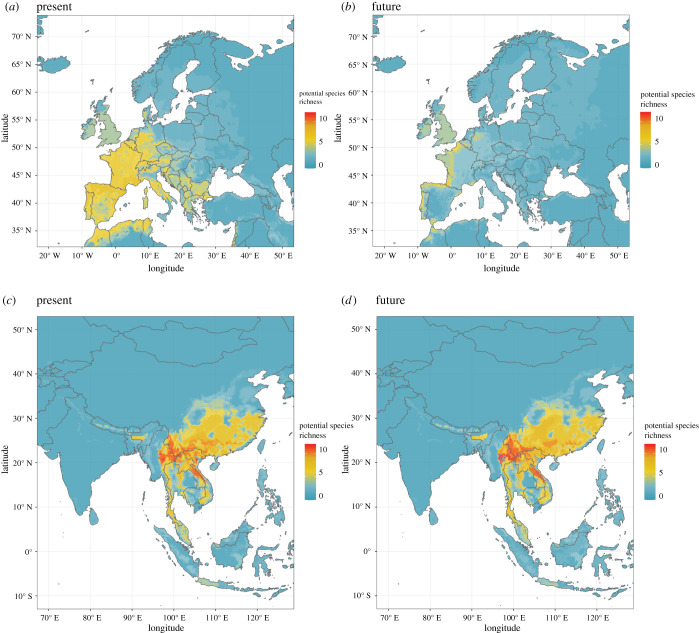
Present (*a,c*) and future (*b,d*) distribution of *Sarbecovirus* bat host species richness, mostly peaking in Europe and Southeast Asia. Projections consider the period 2080–2100 (SSP5-8.5 scenario, BCC-CSM2-MR global circulation model). (Online version in colour.)

The highest number of bat species (i.e. host hotspots) in the present and future projections occurred in Southeast Asia (figure 1 and table 1). The highest values were in Myanmar (13 species), then China, Lao PDR, Thailand and Vietnam (12 species). Area changes are visible in SSP5-8.5 in 2100, and highest richness values are less continuous in the future due to projected species losses (electronic supplementary material, figure S9). The number of contiguous areas with 10 or more estimated species increases from 26 patches in the present to 38 in the future. The overall division index for these hotspot areas increased in future projections from 13.97 to 14.97.

**Table 1.** Highest national maximum *Sarbecovirus* host richness values (i.e. hotspots) predicted through SDMs.

country	subregion	hosts	median sampling rate (mean ± s.d.)
Myanmar	Southeast Asia	13	0.033 (0.053 ± 0.061)
China	East Asia	12	0.024 (0.051 ± 0.064)
Lao PDR	Southeast Asia	12	0.052 (0.063 ± 0.041)
Thailand	Southeast Asia	12	0.087 (0.102 ± 0.067)
Vietnam	Southeast Asia	12	0.086 (0.107 ± 0.074)
Cambodia	Southeast Asia	8	0.112 (0.127 ± 0.076)
India	South Asia	8	0.069 (0.09 ± 0.078)
France	West Europe	8	0.128 (0.144 ± 0.076)
Italy	South Europe	8	0.146 (0.152 ± 0.079)
Malaysia	Southeast Asia	8	0.051 (0.074 ± 0.072)

**Figure 2.**
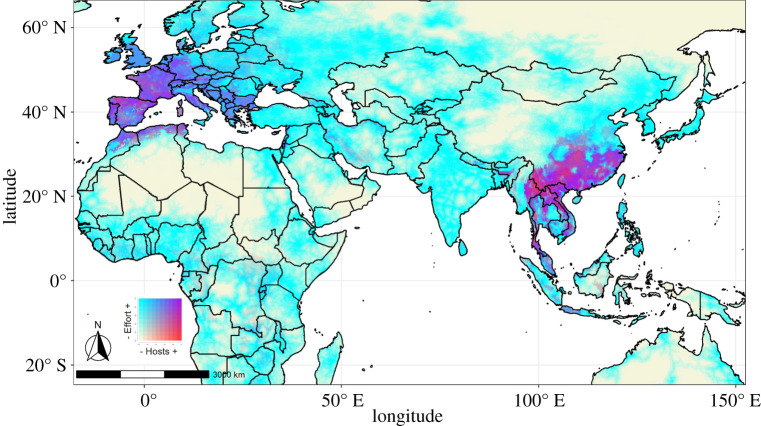
A choropleth bivariate map showing the potential distribution of reported *Sarbecovirus* bat host species and estimated sampling rate calculated for the filtered dataset, according to potential drivers of residual accessibility bias. Darker areas signal high numbers of *Sarbecovirus* hosts, but estimated lower sampling rates driven by accessibility. (Online version in colour.)

From the top 10 countries for maximum potential species richness in a pixel, Italy had the highest estimated sampling rate. Figure 2 shows the interaction between estimated richness of *Sarbecovirus* bat hosts with estimated sampling rates. We highlight areas where the number of species is high and sampling proportion low as future priorities for data collection. Sampling rates were mostly correlated with the distance from roads (electronic supplementary material, figure S10). Overall the estimated sampling rates through accessibility were low for individual locations (min = 0, median = 0.013, mean = 0.045, 3rd q. = 0.065, max = 0.49), even when species are present by ENMs (min = 0, median = 0.08, mean = 0.098, 3rd q. = 0.141, max = 0.446). In Europe, sampling rates show high accessibility bias and better coverage of hosts than other regions, but regions in Southeast Asia are also well sampled, especially eastern coastal areas (purple in figure 2). Highest values for richness were estimated for areas with low sampling rates (electronic supplementary material, figure S11).

**Figure 3.**
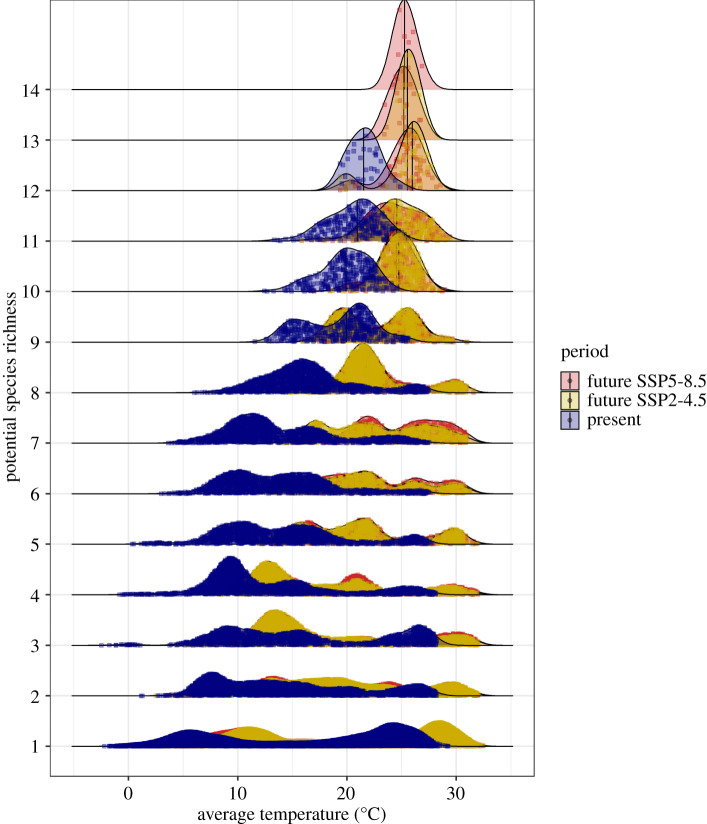
Hotspots of *Sarbecovirus* hosts will be hotter and more concentrated in the future. SSP5-8.5 shows a higher maximum of 14 species in the future, whereas SSP2-4.5 projects 14 species on one occasion, considering BCC-CSM2-MR. Vertical lines represent the median of pixel values and squares represent pixels. (Online version in colour.)

Highest host richness values decline in future SSP5-8.5 projections (figure 3). Average temperature of current hotspots—the few where 13 species are present—is 20.6°C, increasing to 22.7°C in future hotspots (SSP5-8.5, BCC-CSM2-MR in 2100). The hotspot centroid in Southeast Asia is predicted to shift from Myanmar into eastern forests regardless of GCM used. The predicted centroids for highest richness shift from Kat Ku, Myanmar, to denser forest surroundings to the east, only 42 km away, considering BCC-CSM2-MR, or more distantly 373 km further east if we consider the CanESM5 GCM (in the east of Ban Ka Kiak, Lao PDR) by 2100 using SSP5-8.5. Scenarios project increased species richness in locations (figure 3) where temperature is also higher. Temperature increases pose consequences for bat distribution in response to seasonality. Compared to areas with less species, there are smaller averages and ranges for temperature seasonality values in the future host hotspots (electronic supplementary material, figure S12).

Potential ranges for all periods, scenarios and GCMs used are in electronic supplementary material, table S5. Several species showed resilience, such as *Hipposideros armiger*, *H. galeritus* and *H. larvatus*. However, some species with large ranges (electronic supplementary material, figure S13), such as *Rhinolophus ferrumequinum* and *R. affinis*, will probably suffer range contractions (electronic supplementary material, table S6). Population trend data showed that many species do not have a current evaluation (*n* = 20, electronic supplementary material, table S7). Considering the most extreme global warming scenario (SSP5-8.5), most species will suffer range contractions (*n* = 17, 74%), while six may gain area (*n* = 6). For SSP2-4.5, fewer, but still more than half the species will suffer range contraction (*n* = 14, 61%). Overall species estimated occupied areas overlapped less in the future, with higher overlap in SSP2-4.5 in comparison to SSP5-8.5 (electronic supplementary material, figures S14 and S15), however it varies with species (electronic supplementary material, table S8). Range shift trends did not differ across SSPs for most species modelled (*n* = 16, 70%). Most differences pointed to range contractions in SSP5-8.5, while there was a slight expansion in potential ranges in SSP2-4.5 (electronic supplementary material, table S6 and figure S16).

## 4. Discussion

Human-driven habitat change—including through global warming—will alter species distributions, and as a consequence species interactions. Species interactions can have far reaching effects in ecological communities. Parasites and infections that animals carry are often overlooked yet key components of ecological systems [61–63] and host distribution changes may redistribute and alter disease emergence risk. Here, we developed SDMs to determine the drivers of *Sarbecovirus* bat host distributions, identify hotspots of host species richness and model changes in distributions and hotspots under future scenarios. For the species modelled, temperature seasonality and karst were important determinants of geographic distributions, and we identified host hotspots in Europe and Asia. We projected how these hotspots may change under future climate and forest-cover change scenarios, shifting and becoming more fragmented as species' ranges will often contract. We also identified where sampling rates were partially biased, being mainly driven by road accessibility.

Our focal species are insectivorous bats with varying geographical ranges and sensitivity to habitat disturbance [64]. Species responses to climate change can be complex [65,66]. Though some species are resilient (electronic supplementary material, figure S13), *Sarbecovirus* bat hosts are impacted by forest quality and cave disturbance [3], and our projections highlight their overall sensitivity to changes in seasonality. We assume increased temperatures, forest amount and proximity to roost areas will be crucial in driving their future distributions. The implications of host hotspots becoming restricted in the future to warmer tropical, less seasonal environments are unknown [67]. Hosts may have reduced physiological tolerance to higher temperatures and decreasing oscillations in temperature, potentially impacting both their survival and viral dynamics. Concomitant host dispersal to refugia areas and resulting cascading effects may include increased encounters among species that did not overlap before, likely with implications for host switching and pathogen spillover [7]. Zoonotic spillovers, in turn, will be influenced by how people separate themselves from wildlife, especially for viruses with pandemic potential, such as the sarbecoviruses [67]. Wildlife and viral monitoring may be especially relevant in areas where hosts live and humans and potential intermediate hosts use, such as caves and rural areas of Southeast Asia. Monitoring programmes should take into consideration the species-specific role temperature variability and habitat disturbance play in host distribution.

Our findings, therefore, reinforce the need to evaluate differing environment responses even within the same genus. For example, *R. sinicus* lives in montane forests [68], yet *R. affinis* can live in lowland forest, dry forest and disturbed areas [64]. *R. ferrumequinum,* a species ranging from Europe and Northwest Africa to Asia, hibernates during winter in caves, but this varies across the range and with age and sex [64]. Most modelled species are associated with caves (electronic supplementary material, figure S8), with karst availability ranking first or second for seven bat species evaluated in our models in terms of contribution. How karst will change due to mining and land conversion is unclear. Metal mining and limestone quarrying increasingly threaten karst habitats [69] that bats depend upon [3]. We did not model this potential change, but it may reduce suitable habitat and fragment populations.

Remaining primary forests will probably be refugia for many species. The average temperature in the current host diversity hotspots in SE Asia is 20.6°C, potentially increasing to 22.7°C under SSP5-8.5. With hotspots getting hotter, most sarbecovirus hosts’ ranges will contract in the future, following the expected pattern for Southeast Asian bats [70]. Host diversity hotspots will shift to more climatically stable areas where shrinking primary forests remain (electronic supplementary material, figure S9). Suitable areas are lost in northern regions, especially on the China borders. These changes reduce species richness with time in both scenarios used.

We chose a very high GHG emissions scenario as an example here (SSP5-8.5), which is considered a likely scenario [71] evaluated as a possible future in CMIP6, though less likely according to a recent report [72]. Nevertheless, there is high convergence between SSP2-4.5 and SSP5-8.5 hotspot projections (figure 3), though SSP5-8.5 hotspots concentrate more species. In fact SSP2-4.5 is, to some extent, less extreme with fewer range contractions, whereas species are projected to become more spatially concentrated, especially in SSP5-8.5, probably due to a refugia effect [73] since there will be less suitable habitat. We project slight range gains for 2015–2040, possibly due to forest regrowth, which is more likely if international initiatives for reducing deforestation and nature-based solutions succeed [74,75]. After 2040, the high GHG emission scenario projects habitat loss for most species and a shift in the hotspot centroids from Kat Ku, Myanmar, to denser forest surroundings to the east towards Lao PDR. Importantly, this hotspot shift occurs regardless of the global circulation model used.

Our analyses highlight the dynamic and uneven nature of data acquisition. Our pipeline can be easily updated to include new data. Ongoing viral discovery will almost certainly add to the list of bat species testing positive for sarbecoviruses, and many bat species, particularly the Rhinolophidae and Hipposideridae in Africa [76–78] are in need of taxonomic revision. Furthermore, bat species continue to be discovered and described, which could rapidly change conservation assessments. Similar rapid changes happen with viral surveillance, particularly for bats [79,80]. Also, new species descriptions and discoveries can alter sampling bias, since remote areas may be undersampled. More intensive sampling in species-rich, but low sampling effort areas can reduce biases, mainly in areas that are not intensively connected by roads, as identified by our estimates. Despite these challenges, after data curation to reduce sampling bias and autocorrelation, we could still model most species while identifying important biodiversity research shortfalls [81]. The smallest-ranged bats in our dataset did not reach our modelling criteria (electronic supplementary material, figure S7) because of data gaps, which are probably larger for *Sarbecovirus* ecology in their natural hosts (from Wallacean (distribution) to Eltonian (biotic interaction) shortfalls) [16]. By providing a surface of estimated sampling rate, we provide a more realistic scenario for prioritizing sampling of the focal species, as uneven sampling is one of the most common violations of assumption in distribution models [82]. Initiatives to prioritize specific bat viral sampling have been based on phylogeny, expert range definition and viral sharing probabilities [10,83]. We suggest areas with high estimated diversity of hosts and low estimated sampling rates should be a priority for *Sarbecovirus* host studies in the future, such as Indochina and China hotspots (figure 2) [84].

There are conservation implications from our findings. Range contractions are projected for several species, even for species using variable habitats, such as *Rhinolophus pearsonii* (electronic supplementary material, table S4 and figure S5). Most species' populations are currently declining (*n* = 8) or have unknown population trends (*n* = 20, electronic supplementary material, table S6). Local species loss values were almost twice the number of maximum gain (electronic supplementary material, figure S9). Along with climate change mitigation, strategies for maintaining landscape-level habitat connectivity will allow populations to reach refugia and lower extinction risk. This could be done by developing landscape connectivity surfaces that maximize diversity hotspot extensions, with monitoring effective dispersal through genetics [85,86] and population assessments [87].

Our results identify broad regions where bats reported positive for sarbecoviruses most probably occur and co-occur. These hotspots coincide, but are not restricted only to Rhinolophidae diversity hotspots [88] and to hotspots of mammal vulnerability to climate change [89]. Projections suggest that hundreds of new future viral sharing events may occur in Southeast Asia [7]. Novel interactions may be of concern for species survival as pathogens could spread more easily in vulnerable wild populations, which could facilitate epizootics and panzootics [90]. The role of bats as putative reservoirs of different zoonosis-causing agents must be interpreted with care, though [91]. Sarbecoviruses circulating in horseshoe bats might be directly infectious for humans [92,93], or infect other species prior to people [18,94]. Thus, the presence of potential hosts may act as one component of hazard in risk assessments using ecosystem perspectives and multiple drivers [95]. Increased incidence of zoonoses is more likely through human-mediated change of the environment, including climate [96]. Changes in host hotspots may alter disease risk when other changes to human, intermediate domestic or wildlife populations take place [12].

Our future projections assume models using present data perform adequately. However, our models do not account for biotic components that also interfere with suitability, so we are limited to inferences of distribution derived from landscape and climate drivers. Projections will therefore need validation with new data and new predictions. Related, small changes may be relevant for local health and conservation initiatives, and coronavirus hosts are a focus of increasing research [84]. Data change as new hosts are identified [10], host distributions revised, and remote sensing of their drivers updated. We provide a pipeline ready for the inevitable addition of new bat hosts (e.g. [9,80]), which could also be applied for inferring the distribution of potential intermediate hosts for sarbecoviruses. Beyond refining distributional ecology, more work on host characterization will improve our understanding of the role of bats as reservoirs of coronaviruses [84]. Here, we estimated range contractions for most species of bats hosts of sarbecoviruses in response to global changes in climate and forest cover, along with host hotspot shifts. Further evaluations will help inform global change vulnerability assessments [97] and integrative data-modelling steps, in addition to communication of processes involving bats that could benefit One Health [98] and nature-based solutions projects.

## Data Availability

Data provided in the Dryad Digital Repository [34], GBIF [36], and electronic supplementary material [99]. Code provided in GitHub (https://github.com/renatamuy/dynamic) and Zenodo [100].
